# Enormous Hypertrophy of the Spleen

**Published:** 1845-09

**Authors:** 


*Enormous Hypertrophy of the Spleen.*—Dr. Steinbeck observed an enormous hypertrophy and induration of the spleen in a weak country woman, who had frequently suffered during the last ten years from paroxysms of fever, which she used to suppress by fever powders. A hard and unpainful swelling was perceived in the region of the spleen, becoming more and more distinct by the patient's emaciation, which constantly increased. The author first ordered solvent extracts with purgatives internally, and emollient cataplasms, and afterwards emplastr. cicutæ externally. As this treatment, with muriate of ammonia and tartrate of antimony, produced no effects, he ordered iodine externally and internally; within a week a diminution of the tumour was perceptible, and after three weeks the swelling could scarcely be perceived. The hardness of the spleen could only be felt on pressure. The patient continued the use of the iodine, and ultimately took iodide of iron, which caused a great improvement, and restored her to perfect health.—*Ibid, from Preuss. Ver. Zeitung.*

